# TREM2 Regulates Heat Acclimation-Induced Microglial M2 Polarization Involving the PI3K-Akt Pathway Following EMF Exposure

**DOI:** 10.3389/fncel.2019.00591

**Published:** 2020-01-15

**Authors:** Gen-Lin He, Zhen Luo, Ting-Ting Shen, Ze-Ze Wang, Ping Li, Xue Luo, Ju Yang, Yu-Long Tan, Yuan Wang, Peng Gao, Xue-Sen Yang

**Affiliations:** ^1^Department of Tropical Medicine, Army Medical University, Chongqing, China; ^2^Key Laboratory of Extreme Environmental Medicine, Ministry of Education of China, Army Medical University, Chongqing, China; ^3^Department of Nuclear Medicine, Xi’nan Hospital, Army Medical University, Chongqing, China; ^4^Key Laboratory of Medical Protection for Electromagnetic Radiation, Ministry of Education, Army Medical University, Chongqing, China

**Keywords:** EMF, heat acclimation, microglial polarization, TREM2, PI3K-Akt pathway

## Abstract

The function of triggering receptor expressed on myeloid cells-2 (TREM2) has been described within microglia with a beneficial activated phenotype. However, the role of TREM2 underlying microglial phenotypic alterations in the cross-tolerance protection of heat acclimation (HA) against the inflammatory stimuli electromagnetic field (EMF) exposure is less well known. Here, we investigated the TREM2-related signaling mechanism induced by HA in EMF-stimulated N9 microglial cells (N9 cells). We found that EMF exposure significantly increased the production of pro-inflammatory cytokines tumor necrosis factor-α (TNF-α, IL-1β, and IL-6), and the expression of M1 markers (CD11b and CD86); meanwhile, decreased the levels of anti-inflammatory cytokines (IL-4 and IL-10) and the expression of M2 markers (CD206 and Arg1) in N9 cells. Clearly, HA treatment decreased the secretion of TNF-α, IL-1β and IL-6 and the expression of CD11b and CD86, and enhanced the production of IL-4 and IL-10 and the expression of CD206 and Arg1. Moreover, TREM2 esiRNA and selective inhibitor of PI3K clearly decreased anti-inflammatory cytokines production, M2 markers expression, and phosphorylation of PI3K and Akt following HA plus EMF stimulation. These results indicate that TREM2 and PI3K-Akt pathway are involved in the cross-tolerance protective effect of HA in microglial polarization towards the EMF exposure. This finding inspires future studies that aim to explore the non-drug approaches underlying EMF stimulation and other central nervous system (CNS) inflammatory diseases.

## Introduction

Over the past few decades, scientific experiments have reported contradictory results regarding the potential effects of electromagnetic fields (EMFs) on the nervous system (D’Andrea et al., 2003). Several studies have suggested that glial reactivity exhibits a specific sensitivity to EMF exposure (Mausset-Bonnefont et al., 2004; Brillaud et al., 2007; Ammari et al., 2008). As the first line of defense in the central nervous system (CNS), macrophage-like microglia acquire different activation states to modulate immune reactions and the clearance and pro-/anti- inflammatory responses under both physiological and pathological conditions. Although our previous studies revealed the pharmacological outcomes against M1 inflammatory activation in EMF-stimulated N9 microglial cells (N9 cells), approaches for modulating microglial activation states to the M2 phenotype remain poorly understood.

Heat acclimation (HA) is thought to be able to enhance innate cytoprotective pathways against novel stressors *via* cross-tolerance mechanisms (Horowitz, 2017). HA provides neuroprotection against a variety of stressors, including heatstroke (Yi et al., 2017), hyperoxia (Arieli et al., 2003), and traumatic brain injury (Shein et al., 2008). To date, these effects have not yet been studied in response to EMF exposure; however, similar beneficial roles are hypothesized. Additional evidence has revealed that high-energy EMFs have thermal effects (Yang et al., 2010), implying particular roles for heat resistance of acclimation following EMF exposure. It has been reported that HA enhances the presence of microglia with properties of the M2 phenotype, which express the neurotrophin brain-derived neurotrophic factor (BDNF; Shein et al., 2008); this linking the beneficial effects of HA on synaptic properties to an enhancement of neuronal survival (Bessis et al., 2007). Importantly, post-experimental traumatic brain injury and, microglial immunoreactivity are also enhanced upon the alleviation of injury in HA-treated mice (Shein et al., 2008). These results suggest that microglia may be involved in HA-induced neuroprotection.

During activation, microglia polarize towards classically activated (type I)/alternatively activated (type II; M1/M2) phenotypes (Mills, 2012), depending on the stimulus and the receptor signals that are triggered. Clearly, the M2 polarization of microglial populations is believed to be neuroprotective to cells and can be observed in HA mice (Shein et al., 2008). M2 microglia produce anti-inflammatory cytokines including IL-4 and IL-10 and express high levels of CD206 and Arg1. In contrast, persistent M1 polarization of microglia is a prominent cause of an excessive production of pro-inflammatory factors, such as tumor necrosis factor-α (TNF-α), IL-1β and IL-6, and M1 markers CD11b and CD86. The phenotype shift may be associated with the regulation of cellular responses by several sensome receptors, including triggering receptor expressed on myeloid cells-2 (TREM2; Hickman et al., 2013). TREM2 signaling increases phagocytosis and the expression of an anti-inflammatory phenotype in microglia (Neumann and Takahashi, 2007; Kleinberger et al., 2014). However, the molecular mechanisms underlying the triggering microglial phenotypic alterations in HA are less well known.

Given the cross-tolerance mechanism of HA and the potential for microglial reaction upon HA, we tested whether HA attenuates M1 polarization (pro-inflammatory cytokines TNF-α, IL-1β and IL-6, and M1 markers CD11b and CD86) and mediates M2 polarization (anti-inflammatory cytokines IL-4 and IL-10, and M2 markers CD206 and Arg1) in EMF-stimulated N9 cells. Moreover, we utilized pharmacological and enzymatically prepared siRNA (esiRNA) to investigate the molecular mechanisms that regulate the microglial phenotype by HA in EMF-stimulated N9 cells. We demonstrated that HA ameliorates the microglial inflammatory response and shifts the microglial phenotype from M1 to M2 *via* the TREM2 pathway following EMF exposure. These results may provide critical information for the importance of HA in neurologic disorders associated with the regulation of microglial phenotypes.

## Materials and Methods

### Cell Culture and Treatment

Immortalized murine microglial N9 cells were grown in Iscove’s modified Dulbecco’s medium (IMDM; HyClone, Logan, UT, USA) supplemented with 10% heat-inactivated fetal bovine serum (FBS; HyClone), 2 mM glutamine, 100 U/ml penicillin, 100 μg/ml streptomycin, and 50 μM 2-mercaptoethanol (Sigma–Aldrich, St. Louis, MO, USA). Resuscitated N9 cells were used within 3–10 passages, and they were passaged every 3 days. One-half of the cell culture medium was replaced with fresh medium every 2 days. The cell culture medium was replaced with serum-free IMDM supplemented with or without LY294002 (a selective inhibitor of phosphatidylinositol 3 kinase (PI3K), 10 μM) or esiRNA (20 nM; Sigma–Aldrich, St. Louis, MO, USA), and the cells were incubated for 30 min or 12 h during the HA process but prior to the end of last cycle. The solvent controls were performed using dimethyl sulfoxide (DMSO; Sigma–Aldrich, St. Louis, MO, USA) and a transient transfection reagent DharmaFECT (GE Healthcare Dharmacon, Lafayette, CO, USA).

### Cellular Heat Acclimation

To culture the cells for a suitable duration for cellular HA, the cells were seeded in 25 cm^2^ T-flasks (3 × 10^6^ cells/flask) and 24-well plates (1 × 10^5^ cells/well) at 37°C in a humidified 5% CO_2_ atmosphere. For HA, we exposed the cells to intermittent temperatures between 37°C and 39.5°C, mimicking the repeated thermal exposure to acquiring HA that has been previously reported (Patton et al., 2018). The cells were maintained in two incubators under a 20/4-h cycle of 37°C/39.5°C for 72 h. The culture medium was replaced with fresh preheated medium every 2 days.

### Exposure System

Cells were exposed to a pulsed EMF with 2.45 GHz microwaves in an anechoic chamber, as previously described (Yang et al., 2010). Briefly, cells were exposed to a pulsed EMF in an anechoic chamber. The ambient air temperature inside the anechoic chamber was 25°C to 26°C. A 90 mW EMF pulse was delivered through a rectangular horn antenna connected horizontally to a handset (Philips PM 7320X, Sivers IMA, Kista, Sweden). The pulse width was 2 μs, and the pulse repetition rate was 500 pulses per second. Cells were exposed to 2.45 GHz-pulsed microwaves for 20 min at an average specific absorption rate of 6 W/kg. The medium temperature was maintained at 37°C by circulating heated water through the upper and lower chamber. During sham exposure, cultures were placed in the same conditions but without EMF exposure.

### Enzyme-Linked Immunosorbent Assay (ELISA)

After the designated treatment, cell culture supernatants were collected and stored at −80°C until use for detecting TNF-α, interleukin (IL)-1β, IL-6, IL-4, and IL-10, using the Enzyme-Linked Immunosorbent Assay (ELISA) kits (eBioscience, San Diego, CA, USA) according to the manufacturers’ instructions. Cells were washed three times with ice-cold PBS and resuspended in 100 μl ice-cold PBS. Ten-microliter aliquots of the cell collections were quantified using a cell counter (TC20, Bio-Rad, Hercules, CA, USA).

### Immunoblot Analysis

Cells were washed with ice-cold PBS and scraped in RIPA lysis buffer containing protease and phosphatase inhibitors (Roche, Penzberg, Germany) for 30 min on ice. Lysates were centrifuged at 12,000 rpm for 10 min at 4°C. Whole-cell extracts (80 μg/lane) were separated using 6 or 10% SDS-polyacrylamide gel and then transferred onto PVDF membranes (Bio-Rad, Hercules, CA, USA). The membranes were blocked in PBS with 5% non-fat milk for 1 h and then incubated with their respective primary antibodies against rat anti-mouse CD11b (1:800, AbD Serotec, Oxford, UK), mouse anti-mouse CD86 (1:800, Abcam, Cambridge, MA, USA), and rat anti-mouse TREM2 (1:500, Merck Millipore, Temecula, CA, USA), and with antibodies purchased from Santa-Cruz Biotechnology (Santa Cruz, USA) that recognize mouse anti-mouse CD206 (1:100) and mouse anti-mouse Arg1 (1:100), and from Cell Signaling Technology (Danvers, MA, USA) that recognize rabbit anti-mouse phosphor-PI3K p85 Tyr458 (p-PI3K, 1:800), rabbit anti-mouse phospho-Akt Ser473 (p-Akt, 1:1,000). The membranes were washed four times for 5 min each in Tris-buffered saline Tween-20 (TBST) and then incubated with horseradish peroxidase (HRP)-conjugated secondary antibodies (ZsBio) for 1 h at room temperature. After incubation, the membranes were reacted with enhanced chemiluminescence reagent (Bio-Rad, Hercules, CA, USA), and the signal was detected using a ChemiDoc MP gel imaging system (Bio-Rad, Hercules, CA, USA). β-actin (1:1,000; Sigma–Aldrich, St. Louis, MO, USA) was used as an internal control. Relative band densities were determined by densitometric analysis using Image Lab software (Bio-Rad, Hercules, CA, USA).

### siRNA Transfection

Cells were seeded in 25 cm^2^ T-flasks or 24-well plates. Following 60 h of HA, the cells were transfected with an esiRNA (Sigma–Aldrich, St. Louis, MO, USA) targeting TREM2 (20 nM) or a control esiRNA using DharmaFECT (GE Healthcare Dharmacon, Lafayette, CO, USA) in Opti-MEM (Invitrogen Life Technologies, CA, USA) following the manufacturer’s protocol. After culturing, the effects of esiRNA on protein expression were analyzed using Western blotting.

### Immunofluorescence

Cells were grown on coverslips in 24-well plates. After being fixed and permeabilized, the cells were blocked with goat serum (ZsBio) for 20 min at room temperature and washed three times in PBS. The cells were incubated with rat anti-mouse TREM2 (Merck Millipore), rabbit anti-mouse phosphor-Ser473-Akt (Cell Signaling Technology, Danvers, MA, USA), and/or mouse anti-mouse CD206 (Santa Cruz) antibodies at 37°C for 1 h. After being washed, the slides were incubated for 1 h at 37 °C with rabbit anti-mouse AlexaFluor 488 (Life Technologies, Carlsbad, CA, USA), goat anti-rat CF568 (Sigma–Aldrich, St. Louis, MO, USA), and chicken anti-rabbit CF633 antibodies (Sigma–Aldrich, St. Louis, MO, USA) in the dark. The slides were then washed and mounted with an aqueous-based anti-fade mounting medium. Images of stained cells were captured using an LSM 780 confocal laser-scanning microscope (Carl Zeiss GmbH, Jena, Germany).

### Statistical Analysis

Statistical analyses were performed using SPSS PASW Statistics 18.0 software (SPSS, Inc., Somers, NY, USA). Each experiment was repeated a minimum of three times, and the data are expressed as the means ± SEM. Significant differences between the groups were assessed by one- or two-way ANOVA followed by Tukey’s test. Statistical significance was established at *P* < 0.05, unless otherwise indicated.

## Results

### Heat Acclimation Regulates Microglial Polarization in EMF-Stimulated N9 Cells

**Figure 1:**
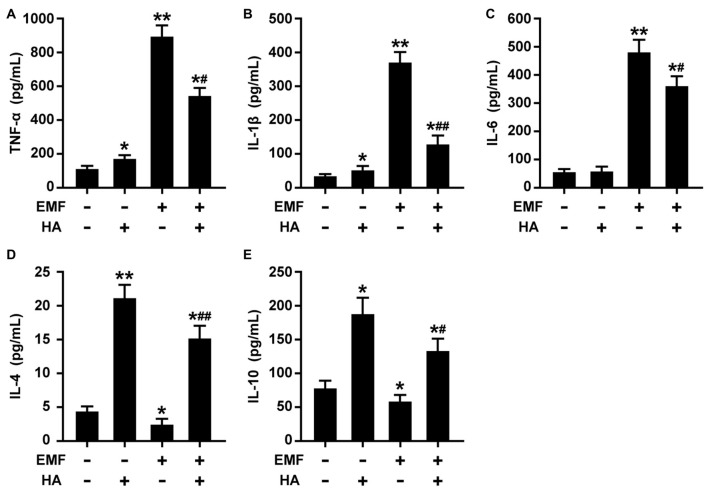
Heat acclimation (HA) regulates the production of pro-/anti-inflammatory mediators in electromagnetic field (EMF)-stimulated N9 cells. N9 cells were pretreated with or without a 72-h HA process (20/4-h cycle of 37°C/39.5°C) and then exposed to 2.45-GHz EMF or sham-exposed for 20 min. Enzyme-Linked Immunosorbent Assay (ELISA) of tumor necrosis factor-α (TNF-α) **(A)**, IL-1β **(B)**, IL-6** (C)**, IL-4 **(D)**, and IL-10 **(E)** production in the cell culture supernatants of N9 cells with or without HA pretreatment 12 h after EMF exposure. Data are presented as means ± SEM of three independent experiments. *P < 0.05, **P < 0.01 vs. the sham-exposed control group; ^#^*P* < 0.05, ^##^*P* < 0.01 vs. the EMF-exposed group.

**Figure 2:**
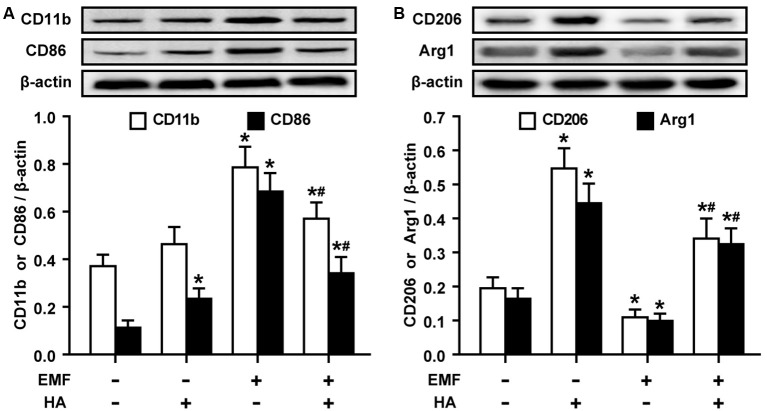
HA regulates the expression of cell surface markers in EMF-stimulated N9 cells. N9 cells were pretreated with or without a 72-h HA process (20/4-h cycle of 37°C/39.5°C) and then exposed to 2.45-GHz EMF or sham-exposed for 20 min. Levels of cell surface marker CD11b and M1 marker CD86 **(A)**, and M2 markers CD206 and Arg1** (B)** in total cell lysates were analyzed using Western blotting, and the corresponding densitometric analyses were represented. Data are presented as means ± SEM of three independent experiments. *P < 0.05 vs. the sham-exposed control group; ^#^*P* < 0.05 vs. the EMF-exposed group.

The production of pro-/anti-inflammatory mediators and M1/M2 cell surface marker expression were determined to test whether HA regulates microglial polarization in EMF-stimulated N9 cells. Consistent with previous studies of neuroprotective functions after HA (Shein et al., 2007a), we found that HA was shown to not alter cell state, and suppressed the production of pro-inflammatory cytokines and increased the production of anti-inflammatory cytokines in N9 cells 12 h after a 20-min EMF exposure (Figure 1). The 20-min EMF treatment was identified as a threshold condition representing the time of duration beyond which cytotoxicity significantly increases as explained in a previous study (He et al., 2016). In addition, based on the previous time effect experiments of EMF-induced pro-inflammatory responses (Yang et al., 2010; He et al., 2014), the threshold 12-h recovery time after EMF exposure with prominent pro-inflammatory activity was employed in the present study. Clearly, ELISA detection indicated that EMF exposure resulted in increased levels of TNF-α (Figure 1A), IL-1β (Figure 1B), but not IL-6 (Figure 1C), and decreased levels of IL-4 (Figure 1D) and IL-10 (Figure 1E). In contrast, compared to the EMF-exposed group, the production of TNF-α, IL-1β, and IL-6 dramatically decreased, and the production of IL-4 and IL-10 significantly increased in EMF-stimulated N9 cells pretreated with HA (HA-plus-EMF-stimulated N9 cells). Compared to the non-HA and sham exposed-control cells, HA-treated N9 cells exhibited slightly increased levels of TNF-α and IL-1β and a significant increase in IL-4 and IL-10. Moreover, Western blotting analysis showed that the expression of M2 markers CD206 and arginase 1 (Arg1) was significantly increased in HA-treated N9 cells (Figure 2B). Consistent with the weak induction of pro-inflammatory mediators by HA treatment, the expression of M1 marker CD86 was also slightly increased in HA-treated N9 cells, compared to the non-HA and sham exposed-control cells (Figure 2A). It was also found that EMF exposure significantly increased the expression of cell surface marker CD11b and M1 marker CD86 (Figure 2A) and decreased the expression of CD206 and Arg1 (Figure 2B), and these effects were reversed by HA pre-conditioning. These results indicate the ability of HA treatment to regulate microglial phenotypes in response to EMF stimulation.

### Alterations in TREM2 and Akt Are Associated With the Modulatory Effects of HA

**Figure 3:**
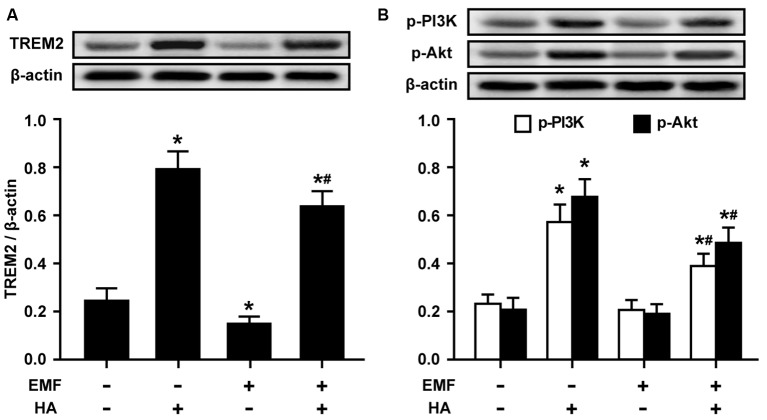
Effects of HA on the expression of triggering receptor expressed on myeloid cells-2 (TREM2) and the phosphorylation of PI3K and Akt in EMF-stimulated N9 cells. N9 cells were pretreated with or without a 72-h HA process (20/4-h cycle of 37°C/39.5°C) and then exposed to 2.45-GHz EMF or sham-exposed for 20 min. Levels of TREM2 **(A)**, and phosphorylation of PI3K and Akt **(B)** in total cell lysates were analyzed using Western blotting, and the corresponding densitometric analyses were represented. Data are presented as means ± SEM of three independent experiments. *P < 0.05 vs. the sham-exposed control group; ^#^*P* < 0.05 vs. the EMF-exposed group.

Given the essential role of Akt phosphorylation in HA-induced neuroprotection (Shein et al., 2007b) and the important protective functions of cell phenotype activation by microglial TREM2 (Piccio et al., 2007), we examined the levels of TREM2 expression and PI3K and Akt phosphorylation in EMF-stimulated N9 cells pretreated with HA. Western blotting analysis showed that the expression of TREM2 was significantly decreased in the EMF-stimulated N9 cells (Figure 3A). HA treatment dramatically augmented TREM2 expression 12 h after EMF exposure (Figure 3A). Subsequently, phosphorylation of PI3K and Akt were quite low both in the sham-exposed control cells and in the EMF-stimulated N9 cells (Figure 3B). Similar to TREM2 expression, HA treatment clearly enhanced the phosphorylation of PI3K and Akt, compared to the control cells (Figure 3B). Moreover, HA treatment significantly reversed the low level of PI3K and Akt phosphorylation 12 h after EMF exposure (Figure 3B). These findings suggest that TREM2 expression and Akt phosphorylation play important roles in the regulatory effects of HA in EMF-stimulated N9 cells.

### esiRNA TREM2 Abolishes the Amelioration of Microglial M2 Polarization by HA

**Figure 4:**
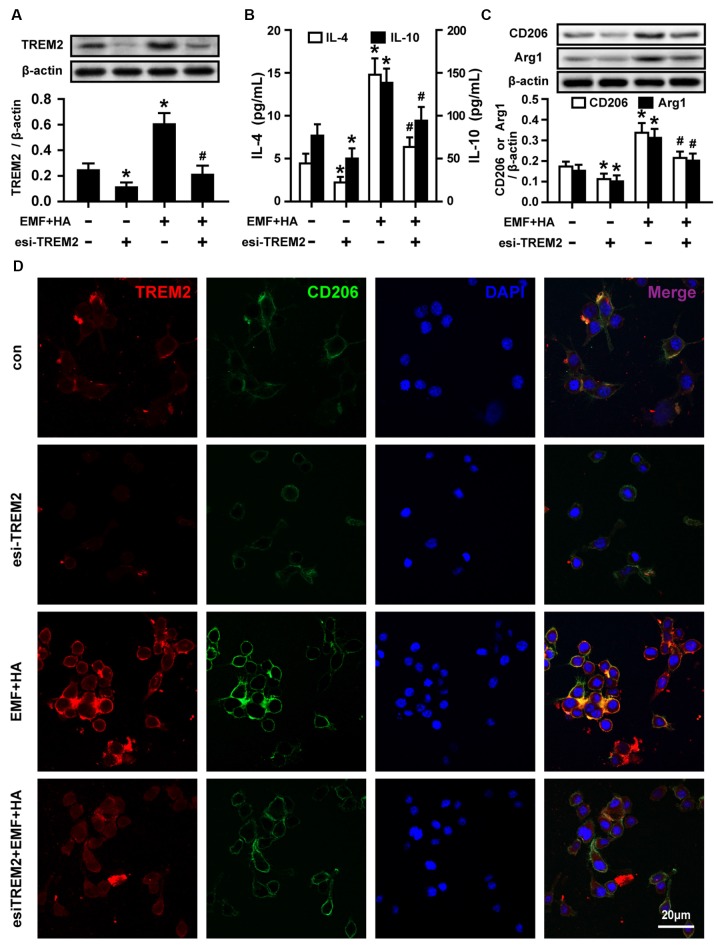
Effects of TREM2 esiRNA on M2 microglial phenotype regulation in EMF-stimulated N9 cells with HA preconditioning. N9 cells were transfected with or without TREM2 esiRNA (20 nM) at 60 h during the uncompleted 72-h HA and then continuously cultured 12 h to complete the HA process and the 24 h transfection of TREM2 esiRNA. Western blotting quantification of TREM2 **(A)**, and M2 markers CD206 and Arg1 **(C)**, and ELISA of anti-inflammatory cytokines IL-4 and IL-10** (B)** production of in either control or HA-plus-EMF-treated N9 cells with or without TREM2 esiRNA. Data are presented as means ± SEM of three independent experiments. *P < 0.05 vs. the non-HA and sham-exposed control group; ^#^*P* < 0.05 vs. the HA-plus-EMF-exposed group. **(D)** Confocal immunofluorescence microscopy was performed on cultures that were immunoreacted with antibodies against TREM2 and CD206 with esi-TREM2 treatment in HA-plus-EMF-treated N9 cells. Scale bar: 20 μm.

To further confirm the involvement of TREM2 in M2 microglial phenotype regulation, esiRNA was used to evaluate the effect of silencing TREM2 on microglial M2 polarization in EMF-stimulated N9 cells with HA preconditioning. The knockdown of TREM2 by a specific esiRNA was confirmed 24 h post-transfection, and the effectiveness of TREM2 inhibition was confirmed by Western blotting. TREM2 esiRNA suppressed the expression of TREM2 in both control and HA-plus-EMF-treated N9 cells (Figure 4A), which were incubated with esi-TREM2 12 h during the HA process but prior to the end of HA and 12 h after a 20-min consequent EMF exposure. The secretion of IL-4 and IL-10 was dramatically decreased in both control and HA-plus-EMF-treated N9 cells treated with esi-TREM2 (Figure 4B). Consistent with the inhibition of anti-inflammatory mediators by esi-TREM2, it was found that the levels of the M2 markers CD206 and Arg1 were significantly attenuated by esi-TREM2 treatment in both control and HA-plus-EMF-treated N9 cells (Figure 4C). Similarly, confocal microscopy provided further evidence of the expression of TREM2 and CD206 and showed a strong increase in fluorescence intensity in HA-plus-EMF-treated N9 cells (Figure 4D). In contrast, esi-TREM2 abrogated the increased expression of TREM2 and CD206 evoked by HA plus EMF treatment (Figures 4C,D). These results indicate that TREM2 esiRNA abolishes the M2 microglial phenotype induced by HA in EMF-treated N9 cells and that TREM2 may be the intracellular signal molecule that mediates M2 polarization of microglia during HA.

### Inhibition of Akt Blocks the TREM2-Mediated Amelioration of Microglial M2 Polarization

**Figure 5:**
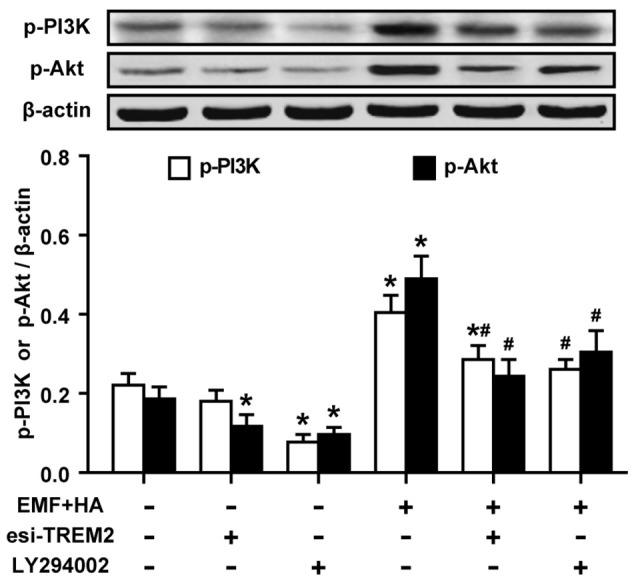
Effects of TREM2 esiRNA and the PI3K inhibitor LY294002 on the phosphorylation of PI3K and Akt in EMF-stimulated N9 cells with HA preconditioning. N9 cells were transfected with or without TREM2 esiRNA (20 nM) at 60 h during the uncompleted 72-h HA and then continuously cultured 12 h to complete the HA process and the 24 h transfection of TREM2 esiRNA. Alternatively, N9 cells were treated with or without LY294002 (10 μM) for 30 min prior to the end of the 72-h HA process. Levels of PI3K and Akt phosphorylation in total cell lysates were analyzed using Western blotting, and the corresponding densitometric analyses were represented. Data are presented as means ± SEM of three independent experiments. *P < 0.05 vs. the non-HA and sham-exposed control group; ^#^*P* < 0.05 vs. the HA-plus-EMF-exposed group.

**Figure 6:**
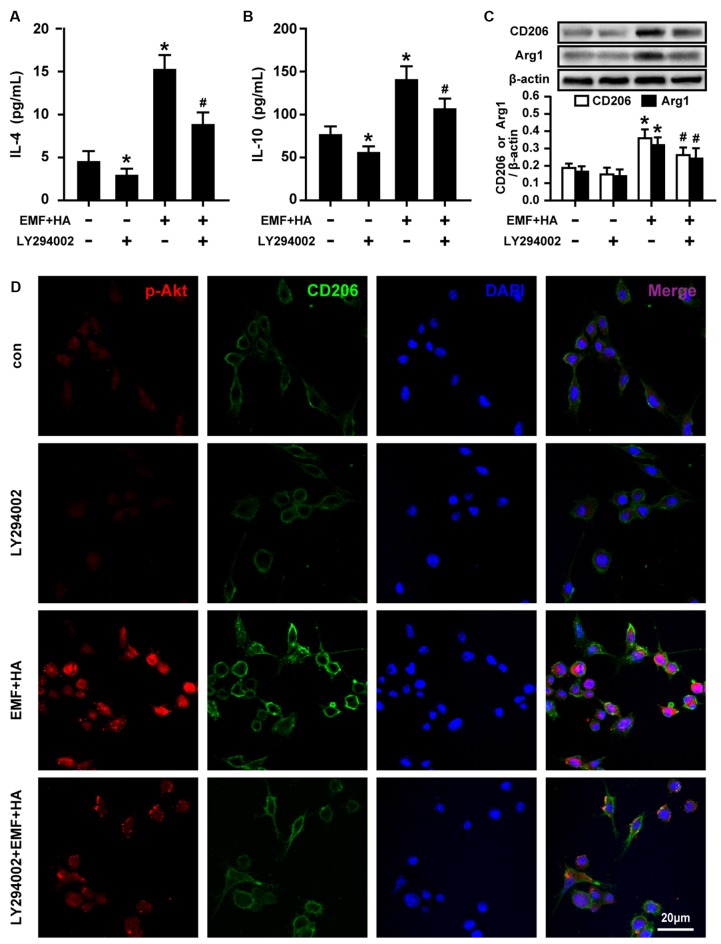
Effects of LY294002 on M2 microglial phenotype regulation in EMF-stimulated N9 cells with HA preconditioning. N9 cells were treated with or without LY294002 (10 μM) for 30 min prior to the end of the 72-h HA process. ELISA of anti-inflammatory cytokines IL-4 **(A)** and IL-10 **(B)** production of in either control or HA plus EMF-treated N9 cells with or without LY294002 pretreatment. **(C)** The expression of CD206 and Arg1 were determined, and the corresponding densitometric analyses were represented. Data are presented as means ± SEM of three independent experiments. *P < 0.05 vs. the non-HA and sham-exposed control group; ^#^*P* < 0.05 vs. the HA-plus-EMF-exposed group. **(D)** Confocal immunofluorescence microscopy was performed on cultures that were immunoreacted with antibodies against p-Akt and CD206 with LY294002 treatment in HA-plus-EMF-treated N9 cells. Scale bar: 20 μm.

To reveal the regulatory signaling effector underlying the TREM2-mediated amelioration of microglial M2 polarization by HA, PI3K and Akt phosphorylation were measured upon treatment with TREM2 esiRNA. TREM2 esiRNA treatment was found to attenuate the phosphorylation of PI3K and Akt in HA-plus-EMF-treated N9 cells (Figure 5). To further investigate whether the mechanism by which HA regulates microglial phenotypes is related to the activation of the PI3K-Akt pathway, the PI3K inhibitor LY294002 was used to assess the inhibitory effects of PI3K-Akt on M2 polarization in HA-plus-EMF-treated N9 cells. As expected, LY294002 treatment was found to attenuate the phosphorylation of PI3K and Akt in both control and HA-plus-EMF-treated N9 cells (Figure 5). Moreover, the secretion of IL-4 (Figure 6A) and IL-10 (Figure 6B), and the expression of CD206 and Arg1 (Figure 6C) were significantly decreased by LY294002 treatment in HA-plus-EMF-treated N9 cells. Interestingly, the cells treated with LY294002 alone showed a significant change in IL-4 and IL-10 production but not CD206 and Arg1 expression, compared to that in the control cells. In addition, a similar inhibition of CD206 and p-Akt immunoreactivity was observed upon pre-treatment with LY294002 by microscopy (Figure 6D). These results indicate that microglial TREM2-mediated M2 polarization induced by HA in EMF-stimulated N9 cells is attributable in part to the PI3K-Akt pathway.

## Discussion

Changes in morphology, cell number, surface receptor expression, and the production of cytokines were evaluated to characterize the immunomodulatory effects of microglia against neuronal stress or stimuli (Ransohoff and Perry, 2009; Zhang et al., 2018). In the present study, we observed pro-inflammatory M1 activation and a significant decrease in the microglial M2 phenotype in N9 cells after EMF exposure. These findings are consistent with our previous data, which indicated a pro-inflammatory response and a decrease in phagocytosis in EMF-treated N9 cells (He et al., 2014). Notably, as a beneficial intervention, HA treatment significantly suppressed the secretion of pro-inflammatory cytokines and the expression of M1 markers and increases the secretion of anti-inflammatory cytokines and the expression of M2 markers in N9 cells 12 h after EMF exposure. Studies involving TREM2 esiRNA and the pharmacological inhibition of PI3K-Akt signaling provided evidence that HA improved microglial M2 polarization in EMF-stimulated N9 cells. These results are in agreement with those of other studies that reported altered cytokine expression and Akt phosphorylation in the brains of heat acclimated mice (Shein et al., 2007a,b). Taken together, the results of these studies showed that the PI3K-Akt signaling pathway may ultimately contribute to the TREM2-dependent immunoregulation of microglial M2 polarization by HA treatment in EMF-treated N9 cells.

It is well known that microglia play a crucial role in various neurological and neurodegenerative disorders through their diverse phenotypes and their ability to shift functions (Orihuela et al., 2016). Over the past few decades, a strong glial reaction in different brain regions has been observed in several studies of EMF effects (Mausset-Bonnefont et al., 2004; Brillaud et al., 2007). In previous work, we demonstrated that EMF may act primarily as a stress inducer and mediate pro-inflammatory activation and a decrease in the phagocytic activity of microglia (He et al., 2014). Consistently, in the present study, we observed an increased production of pro-inflammatory cytokines (TNF-α, IL-1β, and IL-6), cell surface marker CD11b, and M1 marker CD86 and detected decreased anti-inflammatory cytokines (IL-4 and IL-10) and M2 markers (CD206 and Arg1) in EMF-stimulated N9 cells. Notably, the present data and our previous results indicate that EMF exposure is related to an increased risk of neuroinflammatory processes and impaired microglial clearance and is thus associated with an over-activated phenotype of microglia. In support of this, a recent study reported the amelioration of neural injuries *via* shifting microglia from a pro-inflammatory to an anti-inflammatory phenotype as a result of EMF exposure (Zhang et al., 2019). However, extending the methodological and pharmaceutical focus regarding the control of M1/M2 marker expression as well as pro-/anti-inflammatory cytokine production in EMF-stimulated microglia remains a central challenge. Thus, an effective therapeutic option might reveal the detailed mechanisms of the neuroprotective effect of the microglial M2 phenotype upon EMF exposure.

The associated cellular and molecular protective mechanisms of heat-induced tolerance/adaptations attained during HA may be responsible for the attenuation of adverse injury and enhancement of cytoprotective signals (Horowitz, 2017). Remarkably, we delineated the strong immunoregulatory capabilities of HA against the pro-inflammatory M1 phenotype evoked by EMF exposure in N9 cells. In support of this, HA rats and mice display improved functional recovery and reduced cerebral edema formation after closed head injury (Shohami et al., 1994; Shein et al., 2005). Moreover, the neuroprotective role of HA has been reported to be associated with altered levels of cytokines and BDNF of microglia (Shein et al., 2008). Apart from this, in this study, a similar protective M2 phenotype of microglia characterized by increases in IL-4 and IL-10 production and the expression of the M2 markers CD206 and Arg1 is also found in EMF-stimulated N9 cells pre-treated with HA. Taken together, our results combined with those of previous studies suggest, in part, the involvement of microglial reactions in the regulatory role of HA in EMF-stimulated N9 cells. Notably, the regulation of microglial polarization has been clearly reported to possess potential immunoregulatory capabilities using numerous natural compounds. Recently, Schisantherin A exhibits anti-inflammatory effects *via* the activation of the ERK-Nrf2 pathway in lipopolysaccharide (LPS)-activated BV-2 microglial cells (Li et al., 2018). Yang et al. (2017) demonstrated that resveratrol reduces inflammatory damage and promotes microglial polarization to the M2 phenotype *via* PGC-1α in LPS-activated BV-2 cells. In addition, fingolimod, an agonist of the S1P (sphingosine-1-phosphate) receptor, primes microglial M2 polarization *via* the STAT3 pathway in mice with white matter ischemia (Qin et al., 2017). Additionally, malibatol A has been found to regulate microglial M1/M2 polarization through the activation of PPARγ in experimental middle cerebral artery occlusion mice (Pan et al., 2015). However, external HA as a non-drug physical intervention to trigger microglial phenotypic alterations challenges various signaling proteins and receptors. The microglial sensome includes some highly enriched gene transcripts, even including unknown transcripts of sensomes that have not been previously described in microglia (Hickman et al., 2013). Thus, the underlying mechanism of microglial polarization due to HA pre-treatment following EMF exposure remains to be fully clarified.

Triggering receptor expressed on myeloid cells 2 (TREM2) is an innate regulatory receptor of microglia that is linked to the inhibition of inflammatory responses and the potentiation of phagocytic effects in the CNS (Takahashi et al., 2005; Zhong et al., 2015; Xiang et al., 2016; Mazaheri et al., 2017). In this study, the understanding of microglial immunomodulatory phenotypes was expanded, and HA pre-treatment was also found to induce a robust increase in TREM2 expression, which is in line with the elevation of Akt phosphorylation. Additionally, a few genes associated with the direct regulation of microglial TREM2 were detected. A recent study by Boza-Serrano et al. (2019) demonstrated the role of the direct interaction between TREM2 and gal3 in driving microglial activation in Alzheimer’s disease (AD). Zhong et al. (2015) reported that TREM2-DAP12 interactions are required for signal transduction that triggers microglial polarization and consequent cytokine release (Zhao et al., 2018). Additionally, increasing evidence has demonstrated that APOE isoforms interact with TREM2 in association with AD pathogenesis (Wolfe et al., 2018). Although the immunomodulatory mechanisms of TREM2 action and the associated effects of intervening in this mechanism have been widely described, the exact mechanism by which TREM2 regulates microglial polarization during EMF exposure remains to be solved.

In the present study, we demonstrated that microglial TREM2-mediated M2 polarization induced by HA is attributable in part to the phosphorylation of PI3K and Akt in EMF-stimulated N9 cells. In support of these findings, PI3K-Akt signaling has been reported to be required for TREM2-mediated immune modulation (Sun et al., 2013; Zhu et al., 2014; Wolfe et al., 2018). Moreover, increasing evidence has indicated a potential direct link between M2 polarization and the activation of mTOR *via* the PI3K-Akt pathway (Weichhart and Saemann, 2008; Byles et al., 2013; Mercalli et al., 2013). In addition, the PI3K-Akt pathway has been shown to elicit the anti-inflammatory M2-like effects of insulin on macrophage phenotype switching in a diabetic rat model and human monocytic THP-1 cells (Yu et al., 2019). Other investigators, however, have argued that PI3K-Akt is essential in LPS-activated M1 inflammatory microglia (Cianciulli et al., 2016). Thus, the role of PI3K-Akt pathway in stress responses is controversial. This discrepancy may be partly attributable to the intensity, frequency, and duration of the different experimental stimuli. Therefore, it is clear that the mechanism of microglial polarization to stress is complex and multifaceted. Despite the immunomodulatory effect on microglial phenotypes by HA in our study, the TREM2-mediated PI3K-Akt signaling pathway represents a potential therapeutic target for enhancing M2 microglial polarization following EMF exposure.

## Conclusion

Our data provided detailed information regarding the pro-/anti-inflammatory cytokines and M1/M2 markers involved in the regulation of microglial phenotypes in EMF-activated N9 microglial cells. Additionally, we also provide evidence to suggest that TREM2 and Akt signaling, at least in part, trigger microglial M2 polarization through HA in EMF-stimulated N9 cells. Regardless of the detailed mechanism by which Akt is involved in TREM2-mediated M2 polarization, HA as a non-drug treatment permits an extended effort to allow for more cytoprotective signals related to microglial immune responses in neurodegenerative diseases.

## Data Availability Statement

All datasets generated for this study are included in the article.

## Author Contributions

X-SY and G-LH contributed to the conception, design of the study, designed and performed the experiments, analyzed the data, and wrote the article. YW and PG provided the EMF exposure system. PL and Y-LT performed the esiRNA and pharmacological intervention approach. G-LH, Z-ZW, and JY carried out the confocal double-label immunofluorescence assays. ZL, T-TS, and XL contributed to ELISA and western blotting assays. All authors read and approved the final manuscript.

## Conflict of Interest

The authors declare that the research was conducted in the absence of any commercial or financial relationships that could be construed as a potential conflict of interest.
